# First-episode vs recurrent nonspecific neck pain: clinical characteristics, recovery, and the impact of pain severity on well-being and functionality

**DOI:** 10.1097/PR9.0000000000001259

**Published:** 2025-04-03

**Authors:** Martine J. Verwoerd, Harriet Wittink, Francois Maissan, Sander M.J. van Kuijk, Rob J.E.M. Smeets

**Affiliations:** a Research Group Lifestyle and Health, Utrecht University of Applied Sciences, Utrecht, the Netherlands; b Department of Clinical Epidemiology and Medical Technology Assessment, Maastricht University Medical Centre, Maastricht, the Netherlands; c Department of Rehabilitation Medicine, Research School CAPHRI, Maastricht University, CIR Clinics in Revalidatie, Eindhoven, the Netherlands; d Pain in Motion International Research Group (PiM)

**Keywords:** Neck pain, Pain severity, Recurrence, Classification

## Abstract

**Objective:**

This study (1) compared clinical characteristics between patients with first-episode and recurrent nonspecific neck pain (NSNP), and (2) examined differences in daily activities and psychological well-being between patients with minimal pain (1–2 numeric pain rating scale) and moderate to severe pain (≥3 numeric pain rating scale) at 6 weeks postpresentation.

**Methods:**

The study was conducted across 30 primary physiotherapy practices and included patients experiencing a new NSNP episode lasting ≤12 weeks. For the first research objective, baseline data collection encompasses neck pain symptoms, prior conditions, and work-related, general, psychological, and behavioral factors. In addition, pain intensity was measured at 6-week, 3-month, and 6-month follow-ups. For the second objective, pain intensity, psychological factors, and disability were assessed.

**Results:**

No clinically meaningful differences were observed in clinical characteristics or recovery rates between patients with first-episode and recurrent NSNP. However, meaningful differences were found in disability, patient concerns, and self-efficacy between minimal and moderate to severe pain groups at 6 weeks. Patients with higher pain intensity had higher disability scores (1.33-point [SD 0.84–1.81]) difference on a 0 to 7 scale, lower self-efficacy (−1.25-point [SD −1.84 to −0.65]) difference on a 0 to 12 scale, and greater concerns (1.87-point [SD 1.21–2.52] difference on a 0 to 10 scale).

**Conclusion:**

No clinically meaningful differences in clinical characteristics or pain recovery rates exist between first-episode and recurrent NSNP. However, significant and clinically meaningful differences exist in the impact of pain severity on daily activities, patient concerns, and self-efficacy.

## Abstract 2

Supplemental Digital Content is Available in the Text.

Meaningful differences in daily activities, patient concerns, and self-efficacy between patients with different pain intensities. No differences in clinical characteristics first-episode and recurrent nonspecific neck pain patients.

## 1. Introduction

Effective classification of patients with nonspecific neck pain (NSNP) is important for optimizing intervention strategies, improving prognostic accuracy in clinical decision-making, and facilitating clinical research by studying homogeneous patient groups.^4^ Existing treatment-based classification systems are diverse^16^ and often lack accuracy.^5,11,28^ In the International Classification of Diseases - 11 (ICD-11), the International Association for the Study of Pain categorizes chronic pain into secondary pain, which is related directly to a disease, and primary pain, which is considered a disease in its own right.^22^ Chronic primary musculoskeletal pain (CPP) is defined as pain persisting or recurring over 3 months, causing significant emotional distress or functional disability without direct attribution to a known disease and that cannot be better accounted for by another chronic pain condition.^19^

The ICD-11 highlights the need for a multimodal approach that integrates psychological, social, and biological factors in assessing and treating chronic pain.^19,22^ It also recommends optional specifiers for pain intensity, pain-related interference with daily functioning, and pain-related distress aligned with WHO severity stages.^13^ These are measured using a numeric pain rating scale (NPRS) and subsequently translated into severity stages: “mild (NPRS: 1–3),” “moderate (NPRS: 4–6),” and “severe (NPRS 7–10),” to enhance clinical communication and research interpretability.^27^

However, significant variability remains in diagnostic, treatment, and prognostic studies related to patient sampling and subgrouping in chronic pain research, which complicates unbiased comparisons across individual studies. This variability raises concerns about whether the definition of CPP sufficiently addresses these challenges. The optional nature of pain intensity, emotional distress, and disability ratings in the diagnosis of CPP also does not sufficiently resolve these issues.

Moreover, although the definition of CPP excludes acute pain, it encompasses recurrent pain, which often presents as episodic flare-ups with significant variability in symptom severity. These patients experience periods of high pain intensity that severely affect emotional well-being and functionality, alternating with pain-free or minimal-pain periods (<3 NPRS) with minimal impact.^12,17,19,22,27,35^ Although this group may meet the CPP criteria during flare-ups, they do not during minimal-pain periods, leading to potential misclassification in pain research. This variability introduces heterogeneity in study populations, which can bias treatment effect results. Moreover, many studies prioritize pain intensity as the primary outcome or inclusion criteria, often overlooking these fluctuations and their differential impact on emotional well-being and functionality, further complicating the classification of recurrent pain under the CPP definition.

These concerns ask to clarify the current classification of the ICD-11, on 2 key points: (1) whether recurrent pain should be included in the definition of CPP and (2) whether the pain severity stage should be a mandatory criterion rather than optional when defining CPP.

Given these considerations, our study aimed to explore the distinctions in clinical presentations among NSNP patient groups and examine the impact of pain intensity on daily functioning and psychological well-being. We use the classification from our previous prognostic study to classify patients based on their first and new episodes in a recurrent pattern, as well as their pain intensity scores, to classify a patient as chronic or nonchronic.^31,32^ Therefore, we hypothesized that:

(1) There is no clinically meaningful difference between the clinical presentations (eg, lifestyle, psychological, and behavioral factors) or the 6-week, 3-month, and 6-month recovery rates of patients experiencing their first episode of NSNP and those with a new episode in a recurrent pattern who present themselves in primary physiotherapy practice at baseline.
(2) There is a clinically meaningful difference between groups with differing levels of pain severity (NPRS 1–2 defined minimal pain and NPRS ≥3 defined moderate and severe pain) on daily activities, illness perceptions, and psychological factors measured at 6 weeks follow-up.

## 2. Method

### 2.1. Study design

For this study, we used data from a larger prospective cohort study aimed at identifying prognostic factors for patients experiencing (sub)acute neck pain in primary physiotherapy practices in the Netherlands.^31,32^ This study encompasses a cross-sectional analysis of patient presentations at baseline and the 6-week follow-up time point and a longitudinal observation of patient outcomes over 6 weeks, 3 months, and 6 months. For hypothesis 1, we used baseline data (cross-sectional) and the pain measurements at 6 weeks, 3 months, and 6 months (longitudinal). For hypothesis 2 (hypothesis 2), we used data obtained 6 weeks after their first presentation in primary physiotherapy practices (cross-sectional). We used the STROBE statement for cross-sectional and cohort studies as a reporting guideline.^33^

### 2.2. Ethical approval

Ethical approval for this study was obtained from the Medical-Ethical Review Committee of the University Medical Center Utrecht (Protocol Number: 19-766/C). In adherence to privacy standards, all data were processed anonymously, with each participant providing informed consent. Data were securely collected and transmitted using Formdesk, a secure data management system.^15^

### 2.3. Setting

Potential participants were selected from 30 private physiotherapy practices that employed 94 physiotherapists. The participants' recruitment extended between January 26, 2020, and August 31, 2022, and the follow-up was completed on March 17, 2023.

### 2.4. Participants

Eligibility for participation was extended to patients presenting with a new episode of (sub)acute nonspecific idiopathic, nontraumatic neck pain. Inclusion criteria were being 18 years or older, having a new onset of neck pain not exceeding 12 weeks, and having neck pain localized between the linea nuchae superior to the scapular spine (see Appendix 1, supplementary materials, http://links.lww.com/PR9/A291). Patients with a history of neck pain were required to have been relatively symptom-free for a minimum of 3 months (NPRS of <3) before the current episode. This threshold was chosen to ensure that the pain intensity during this period would not significantly affect mental health or functionality, thereby effectively excluding patients with chronic pain.^12,34^

Exclusion criteria were previous neck surgery, cervical spine radiculopathy as determined by the Upper Limb Neurodynamic Test 1,^26^ widespread pain as defined in the ICD-11 (diffuse musculoskeletal pain in a minimum of 4 of 5 body regions and at least 3 body quadrants), pain not caused by musculoskeletal origin, and an inability to read or understand the Dutch language.

For hypotheses 2, we only used those participants who 6 weeks after their first presentation at the physiotherapist still experienced neck pain. At this time point, we categorized patients into the minimal pain (1–2 NPRS) and moderate to severe pain groups (≥3).

### 2.5. Variables and measurements

At baseline, we assessed variables to differentiate between patients with first-time and recurrent (sub)acute NSNP. The outcome variable, pain intensity, was assessed at 6 weeks, 3 months, and 6 months (hypothesis 1). At the 6-week follow-up measurement, we assessed disability status, patient perceptions, psychological variables, and sleep quality to analyze differences in these variables between the minimal and moderate–severe pain groups (hypotheses 2). All variables and their measurement moment are summarized in Table 1, and their measurement method in Appendix 2 (see supplementary materials, http://links.lww.com/PR9/A291).

**Table 1:** All variables and their measurement moment for hypotheses 1 and 2.

Hypothesis 1 There is no clinically meaningful difference between the clinical presentation or the 6-week, 3-month, and 6-month recovery rates of patients experiencing their first episode of NSNP and those with a new episode in a recurrent pattern who present themselves in primary physiotherapy practice	
Baseline	Symptoms: Pain intensity at baseline, Duration of Neck Pain, Reported pain in different body regions, Accompanying headache, Disability Lifestyle factors: Physical activity, Smoking, Alcohol, BMI, Sleep quality Psychological and behavior factors: Catastrophizing, Depression, Kinesiophobia, Distress, Hypervigilance, Self-efficacy, Coping Patients' beliefs: Duration beliefs, Concerns, Treatment beliefs, Therapeutic relation, Identity beliefs
Six weeks follow-up	Pain intensity
Three months follow-up	Pain intensity
Six months follow-up	Pain intensity
Hypothesis 2 There is a clinically meaningful difference between groups with differing level of pain severity (NPRS 1–2 defined mild pain, and NPRS ≥3 defined moderate and severe pain) on daily activities, illness perceptions, psychological factors and sleep quality	
Six weeks follow-up	Symptoms: Disability Psychological factors: Catastrophizing, Depression, Kinesiophobia, Distress, Hypervigilance, Self-efficacy Patients' beliefs: Concerns, Therapeutic relation, Identity beliefs

BMI, body mass index; NSNP, nonspecific neck pain.

### 2.6. Study size

In both hypotheses, we examine differences between 2 groups. For hypothesis 1, we assess 30 variables, including pain intensity, functional impairment, psychological factors, and recovery rates, comparing patients with a first episode of neck pain with those with a recurrent episode. For hypothesis 2, we investigate 10 variables across groups stratified by pain intensity. The multiple regression analysis will include a maximum of 9 variables. Sample size calculations were performed for these hypotheses. Our sample size calculation revealed that to achieve 90% power for an independent-samples *t* test to detect a medium effect size (Cohen's *d* of 0.5), we would need approximately 85 patients per group, when testing with an alpha of 0.05. Consequently, the smallest group must at least include 85 patients, assuming a 1:1 ratio. For analyses involving dichotomous and categorical variables, considering 1 to 3 degrees of freedom and adopting Cohen's convention for a medium effect size (w = 0.3), we estimated a required sample size ranging from 165 to 230 participants. The power analysis for the multiple regression analysis, predicting a medium effect size (f^2^ < −0.15), with a power of 80% and a significance level of 0.05, and considering the maximum of 9 variables in the model, indicated a minimum requirement of 108 participants. Therefore, we aimed to include at least as many participants for the most demanding computed sample sizes.

### 2.7. Quantitative variables and statistical methods

We used the R (version 4.2.2) for the sample size calculation and all analyses.^21^ Descriptive statistics to summarize patients' characteristics were recorded in the analysis tables. Incomplete records at baseline and follow-up were imputed by multiple imputations using fully condition specification under the assumption that the data were at least missing at random.^20^ Predictive mean matching was used for continuous variables to draw imputations, and logistic regression was used for categorical variables.

Continuous variables were expressed as mean with standard deviation (SD) and median with interquartile range. Dichotomous and categorical variables were presented using frequencies and percentages. Group differences were analyzed using the independent samples *t* test for continuous variables and Pearson χ^2^ tests for categorical and dichotomous variables. Group differences were visualized with histograms and violin plots. The threshold for statistical significance was set at *P* < 0.05.

Owing to potential confounding factors across different categories, we conducted linear, logistic, and multinomial regression analyses corresponding to continuous, dichotomous, and categorical outcome measures. Multicollinearity was assessed, and variables exhibiting a correlation coefficient higher than 0.8 or a variance inflation factor (VIF) exceeding 5 were excluded from the models.

All variables were adjusted for sex and age and categorized into subgroups based on confounding factors: patient characteristics; symptoms; lifestyle, psychological, and behavioral factors; perception; and pain intensity over time. Multiple regression models accounted for confounding factors in these subgroups. For hypothesis 1, recurrent/nonrecurrent status, and for hypothesis 2, pain intensity groups, were independent variables, along with other potentially confounding factors, alongside potential confounders. The variable of interest, such as distress, was treated as the dependent variable. This approach allowed us to correct for confounding variables, leading to a more accurate assessment of differences and, thereby, enhancing the validity of our results.

In cases where the groups differed significantly on a variable, we compared the group difference with the minimal detectable change (MDC) and the minimal important change (MIC). The MDC indicates changes that fall outside the measurement error of the health status measurement.^8^ The MIC represents the threshold for a minimal within-person change over time, above which patients perceive the changes as meaningful. If the MDC and/or MIC were available, we reported the specific population on which these values were determined. In addition, if the difference was lower than the MIC, we discussed from a clinical perspective whether the difference was clinically meaningful.

## 3. Results

### 3.1. Participants

In 30 Dutch physiotherapy practices, 2,567 patients were evaluated for eligibility, including 603 participants over a 2.5-year period. A total of 1,600 were primarily excluded due to chronic pain, cervical spine radiculopathy, or widespread pain, and 307 declined to participate. Reasons for declining included lack of interest, scheduling issues, or current stress. In addition, 58 individuals did not complete the baseline assessment despite signing informed consent and agreeing to participate. For further details, we refer to Appendix 3 (see supplementary materials, http://links.lww.com/PR9/A291), which contains the study flowchart. The study population included 397 women and 206 men, with a mean baseline pain intensity of 5.9 (SD = 1.9) and a mean disability score of 2.7 (SD = 2.1). Higher scores indicate higher interference of pain with daily activity, where we divided the sum score by the entered items (range of 0–7). The final cohort consisted of 198 individuals (33%) experiencing their first episode of (sub)acute neck pain and 405 (67%) with recurrent (sub)acute neck pain. Psychological variables tended toward a nonnormal distribution with lower scores. Follow-up losses were significant, with 154 participants not submitting forms by 6 weeks, increasing to 224 by 3 months and 231 by 6 months. At 6 weeks, 278 of the 449 responders still experienced neck pain, with a mean pain intensity of 4.2 (SD = 2.0); 67 reported minimal pain (1–2 on the NPRS); while 209 reported moderate to severe pain (≥3 on the NPRS). Of these 278 participants, no data were regarding the variables of interest for the analysis on difference between the 2 pain intensity groups were missing. The variables exhibited correlations below 0.80 and VIF scores below 2.7, both indicators suggesting minimal multicollinearity and thus reducing concerns about its influence on the regression results.

### 3.2. Hypothesis 1

Table 2 summarizes the statistical findings. Across all measured variables, no statistically significant differences were observed between the patients with a first episode of neck pain and those with a new episode of neck pain in a recurrent pattern. Differences in patients' concerns, treatment beliefs, and therapeutic relations showed *P*-values of 0.08, 0.07, and 0.06, respectively. Patient concerns exhibited a mean difference of −0.408 (95% CI: −0.05 to 0.86), treatment beliefs a mean difference of −0.22 (95% CI: −0.01 to 0.45), and therapeutic relation showed a mean difference of −0.216 (95% CI: −0.01 to 0.45), all on a 0 to 10 point scale. When adjusting for various patients' beliefs, age, and sex in the regression analyses, the differences in treatment beliefs and therapeutic relations were smaller. However, concerns was significantly different between groups (*P* = 0.03).

**Table 2:** Group difference recurrent pain and first-episode pain group measured at baseline.

Variables classified in subgroups	Recurrent pain group	First-episode pain group	Mean differences continuous variables (95% CI)	Independent sample *t* tests	Adjusted coefficient (95% CI)	Adjusted *P*
	Mean (SD)	Mean (SD)		*P*		
	Median (IQR)	Median (IQR)				
	Number (%)	Number (%)				
Patients characteristics						
Sex				0.88	0.03 (−0.33 to 0.39)	0.87
1 = male	137 (33.8)	69 (34.8)				
2 = women	268 (66.2)	129 (65.2)				
Age	44.4 (15.9) 42 (25)	44.7 (15.1) 45 (25)	−0.33 (−2.44 to 2.90)	0.84	−1.04 (−3.41 to 1.33)	0.39
Work status (yes/no)	336 (83.0) 69 (17.0)	172 (86.9) 26 (13.1)		0.26	0.32 (−0.22 to 0.88)	0.25
Education				0.30	−0.19 (−0.53 to 0.15)	0.27
Low level of education	222 (54.8)	99 (50)				
High level of education	183 (45.2)	99 (50)				
Symptoms						
Pain intensity at baseline (0–10)	5.88 (1.90) 6 (2)	6.03 (1.80) 6 (2)	−0.15 (−0.16 to 0.46)	0.34	−0.22 (−0.50 to 0.06)	0.12
Duration of neck pain	4.52 (2.92)	4.52 (2.92)	0.00 (−0.50 to 0.50)	0.98	−0.02 (−0.52 to 0.47)	0.92
No. of weeks	4 (4)	4 (4)				
Reported pain in different body regions				0.34	0.18 (−0.18 to 0.54)	0.32
No	136 (33.6)	75 (37.9)				
Yes	269 (66.4)	123 (62.1)				
Accompanying headache						
No	165 (40.7)	84 (42.4)		0.73		
Yes	195 (48.1)	89 (44.9)			0.12 (−0.26 to 0.50) −0.14 (−0.70 to 1.22)	0.53
I had headache(s) before the neck pain	45 (11.1)	25 (12.6)				0.64
Disability (0–7)	2.77 (2.12) 2.3 (3.1)	2.63 (1.92) 2.1 (2.9)	0.14 (−0.48 to 0.20)	0.43	0.21 (−0.11 to 0.52)	0.19
Lifestyle factors						
Physical activity						
Achieving the Dutch healthy exercise norm	144 (35.6)	76 (38.4)		0.56	0.09 (−2.26 to 0.45)	0.60
Not achieving the Dutch healthy exercise norm	261 (64.4)	122 (61.6)				
Smoking (No/Yes)	357 (88.1) 48 (11.9)	174 (87.9) 24 (12.1)		1.00	−0.06 (−0.26 to 0.45)	0.82
Alcohol (No/Yes)	87 (21.5) 318 (78.5)	42 (21.2) 156 (78.2)		1.00	0.01 (−0.42 to 0.42)	0.97
BMI	25.5 (4.33)	25.1 (4.47)	0.43 (−1.22 to 0.33)	0.26	0.42(−0.31 to 1.15)	0.26
Sleep quality						
Sleep quality				0.27	−0.27 (−0.74 to 2.22)	0.21
No negative experience with sleeping	93 (23.0)	37 (18.7)				
Negative experience with sleeping	312 (77.0)	161 (81.3)				
Psychological factors						
Catastrophizing (0–24)	4.53 (4.49) 3 (6)	4.70 (4.70) 3 (6.75)	−0.16 (−0.63 to 0.96)	0.68	−0.29 (−0.88 to 0.29)	0.32
Depression (0–21)	2.58 (3.45) 1 (4)	2.25 (3.16) 1 (3)	0.33 (−0.89 to 0.22)	0.24	0.08 (−0.28 to 0.45)	0.65
Kinesiophobia (11–44)	16.5 (5.08) 15 (8)	16.7 (5.43) 16 (7.75)	−0.26 (−0.65 to 1.17)	0.57	−0.33 (−1.04 to 0.37)	0.35
Distress (0–21)	4.56 (4.14) 4 (6)	4.04 (4.07) 3 (5)	0.52 (−1.22 to 0.18)	0.14	0.23 (−0.22 to 0.67)	0.32
Hypervigilance (0–80)	31.4 (10.9) 31 (14)	30.2 (12.5) 31 (17)	1.17 (−3.21 to 0.88)	0.26	1.09 (−0.54 to 2.72)	0.19
Self-efficacy (0–12)	10.4 (2.29) 11 (2)	10.2 (2.37) 11 (2)	0.13 (−0.53 to 0.26)	0.54	0.13 (−0.22 to 0.48)	0.48
Coping				0.74	−0.07 (−0.53 to 0.37)	0.75
Passive coping	84 (20.7)	38 (19.2)				
Active coping	321 (79.3)	160 (80.8)				
Perception factors						
Duration beliefs (0–10)	4.15 (2.68) 3 (4)	4.06 (2.59) 3 (4)	0.09 (−0.54 to 0.36)	0.70	0.22 (−0.18 to 0.62)	0.28
Concerns (0–10)	3.82 (2.60) 4 (4)	4.23 (2.69) 4 (4)	−0.41 (−0.05 to 0.86)	0.08	−0.46 (−0.86 to 0.06)	0.03*
Treatment beliefs (0–10)	7.72 (2.00) 8 (2)	7.99 (1.57) 8 (2)	−0.27 (−0.02 to 0.57)	0.07	−0.11 (−0.37 to 0.15)	0.42
Therapeutic relation (0–10)	8.73 (1.46) 9 (2)	8.94 (1.28) 9 (2)	−0.22 (−0.01 to 0.45)	0.06	−0.12 (−0.31 to 0.07)	0.22
Identity beliefs (0–10)	6.13 (2.41) 7 (3)	6.07 (2.24) 6 (3)	0.06 (−0.45 to 0.33)	0.76	0.16 (−0.23 to 0.56)	0.41
Outcomes						
Pain at 6 wk	2.54 (2.62) 2 (4)	2.55 (2.62) 2 (5)	−0.01 (−0.44 to 0.46)	0.97	0.05 (−0.33 to 1.15)	0.78
Pain at 3 mo	2.00 (2.63) 0 (4)	2.16 (2.70) 0 (4)	−0.16 (−0.30 to 0.62)	0.49	−0.19 (−0.43 to 0.44)	0.30
Pain at 6 mo	1.23 (2.27) 0 (1)	1.29 (2.50) 0 (1)	−0.06 (−0.36 to 0.48)	0.77	−0.11 (−0.44 to 0.21)	0.49
Chronic pain				0.80	0.77 (−0.21 to 1.84)	0.14
No	362 (89.4)	179 (90.4)				
Yes	43 (10.6)	19 (9.6)				

Significance codes: 0 “***” 0.001 “**” 0.01 “*.”

The adjusted coefficients can be interpreted as the difference between the recurrent and the first-episode pain group.

BMI, body mass index; IQR, interquartile range.

The difference of 0.41 on the concern scale (0–10) was smaller than the MDC of 0.57, which was determined in a chronic obstructive pulmonary disease (COPD) study sample.^6^ The MIC for this factor has not been established.

### 3.3. Hypothesis 2

**Figure 1.**
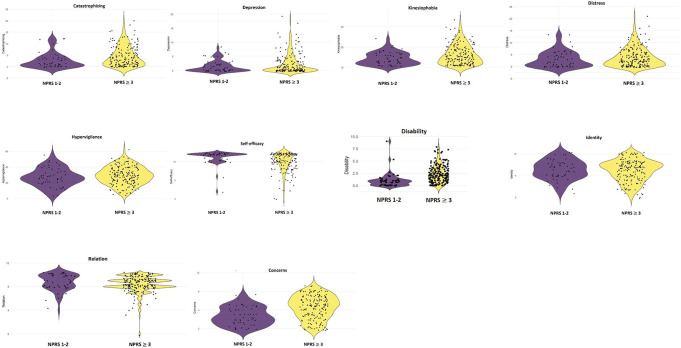
Visualization of the difference in pain severity interference with daily activities, illness perceptions, psychological factors, and sleep quality between pain groups measured at 6 weeks follow-up.

Table 3 and Figure 1 present the results and visual representations. The violin plot demonstrates that disability levels for the moderate to severe pain group were more widely distributed compared with the minimal pain group, where most patients presented with low disability levels. This pattern is also evident for self-efficacy; the moderate to severe pain group displayed a broader distribution in self-efficacy scores, whereas most of the minimal pain group exhibited high pain self-efficacy scores.

**Table 3:** Difference interference pain severity with daily activities, patients' beliefs, and psychological factors at 6 weeks.

Variables classified in subgroups	Mild pain Mean (SD) Median (IQR)	Moderate and severe pain Mean (SD) Median (IQR)	Mean differences (95% CI)	Independent sample *t* tests *P*	Adjusted coefficient (95% CI)	Adjusted *P-* value
Model psychological and behavior factors						
Catastrophizing (0–24)	2.69 (3.36) 1 (4)	4.43 (4.33) 3 (6)	1.74 (0.61 to 2.87)	0.003	0.36 (−0.62 to 1.33)	0.47
Depression (0–21)	1.5 (2.00) 1 (2)	2.52 (3.66) 1 (3)	0.81 (0.06 to 1.56)	0.03*	0.11 (−0.55 to 0.79)	0.73
Kinesiophobia (11–44)	14.4 (3.08) 13 (5)	16.3 (4.76) 15 (7)	1.88 (0.77 to 2.98)	0.001**	0.66 (−0.39 to 1.70)	0.22
Distress (0–21)	3.08 (3.35) 2 (5)	3.89 (3.93) 3 (5)	0.82 (−0.28 to 1.91)	0.14	−0.13 (−0.92 to 0.67)	0.75
Hypervigilance (0–80)	26.7 (11.70) 26 (14.2)	28.8 (14.40) 29 (16.2)	2.11 (−1.54 to 5.75)	0.09	−0.50 (−3.44 to 2.43)	0.73
Self-efficacy (0–12)	11.2 (1.72) 12 (1.25)	9.97 (2.45) 10.5 (3)	−1.25 (−1.84 to −0.65)	<0.001***	−0.86 (−1.55 to −0.17)	0.02*
Model disability						
Disability	1.03 (1.46) 0.9 (1.05)	2.36 (1.78) 2.1 (2.42)	1.33 (0.84 to 1.81)	<0.001***	1.34 (0.81 to 1.88)	<0.001 ***
Model patients' beliefs						
Concerns	2.71 (1.96) 2 (3.25)	4.58 (2.49) 5 (4)	1.87 (1.21 to 2.52)	<0.001***	1.84 (1.10 to 2.57)	<0.001 ***
Therapeutic relation	8.71 (1.38) 9 (2)	8.39 (1.43) 8.5 (1)	−0.43 (−0.76 to 0.11)	0.14	−0.16 (−0.60 to 0.28)	0.47
Identity beliefs	6.35 (2.23) 6.5 (3)	6.18 (2.42) 7 (3)	0.17 (−0.88 to 0.54)	0.64	−0.14 (−0.90 to 0.62)	0.72

Significance codes: 0 “***” 0.001 “**” 0.01 “*.” The adjusted coefficients can be interpreted as the difference between the 2 pain intensity groups (minimal pain to moderate and severe pain).

IQR, interquartile range.

T tests revealed significant differences in the mean scores of catastrophizing, depression, kinesiophobia, disability, and concerns between the minimal and moderate to severe pain group, with the moderate to severe pain group exhibiting higher scores across these factors. Notably, the group with higher pain intensity scored significantly lower on self-efficacy than those with lower pain intensity, with a mean score of −1.25 (95% CI: −1.84 to −0.65) on a 0 to 12 point scale. When adjusting for various psychological factors (see models Table 3), age, and sex in the regression analyses for catastrophizing, depression, and kinesiophobia, the differences between the 2 pain groups became smaller in size and were no longer statistically significant.

T tests and multiple regression analyses consistently showed a significant difference in self-efficacy between the pain intensity groups, with scores of 11.2 (SD 1.72) in the minimal pain group and 9.97 (SD 2.45) in the higher pain intensity group. These differences persisted even after adjustment for various psychological factors, age, and sex. Patients with higher pain intensity also reported significantly greater disability and concern levels, with a mean difference of 1.33 (95% CI: 0.84–1.81) on a 7-point disability scale and a two-point higher concern level on a 10-point scale, remaining significant after adjusting for age and sex. Despite these differences, disability levels were relatively low, with mean scores of 0.99 (SD 1.43) for the minimal pain group and higher for the moderate to severe pain group with mean scores of 2.31 (SD 1.84). Concern levels were scored 2.71 (SD 1.96) on average for those with minimal pain and 4.46 (SD 2.39) for higher pain. The difference was unaffected by adjustments for therapeutic relation, identity beliefs, age, and sex.

There was no MDC available for the short form of the Pain Self-Efficacy Questionnaire (PSEQ). The difference of 1.25 (SD 1.84–0.65) in self-efficacy is higher than the MIC of −0.5 for patients who score high on the 2-item questionnaire, determined in a study population of patients with chronic low back pain.^3^ The group that scores higher on the PSEQ-2 in that study is comparable with our study population scores.

The MDC for the PDI was 17.9 points, which, when adjusted for the total score divided by the number of items completed, corresponds to 2.6 points.^23^ This is higher than our pain groups' 1.33 (SD 0.84–1.81) difference. However, the MIC value of 9.5, corresponding to 1.4 points when divided, is close to our observed difference. These findings are based on a study population with a much higher level of disability among patients with musculoskeletal pain presenting at secondary care facilities.

The difference of 1.87 in illness perception concerns between the pain groups exceeds the SDC of 0.57, indicating a “real difference” between the groups established on a COPD study population.^6^ However, no MIC is available to address the illness perception “concerns.”

## 4. Discussion

This study found few significant differences between the clinical characteristics of patients experiencing a first episode of NSNP and those with a new episode in a recurrent pattern. No differences were observed in their 6-week, 3-month, and 6-month recovery rates in primary physiotherapy care. Despite finding statistically significant differences in the *t* tests, these differences are negated in regression analyses where confounding variables are considered. Only patients' concerns remained significantly different between these groups. However, more significant differences were observed in the interference of pain severity—minimal pain (1–2 NPRS) and moderate to severe pain (≥3 NPRS)—with daily activities (disability), patients' concerns, and self-efficacy. We observed a 1.33-point (SD 0.84–1.81) difference in disability on a 0 to 7 point scale, a 1.25-point (SD −1.84 to −0.65) difference in self-efficacy on a 0 to 12 scale, and a 1.87-point (SD 1.21–2.52) difference on patients' concerns a 0 to 10 scale. Whether these significant results are clinically meaningful will now be discussed.

The absence of an established MDC for the Pain Self-Efficacy Questionnaire-2 raises concerns about accurately measuring the observed 1.25-point difference in self-efficacy at the group level. In addition, the PSEQ-2's significant ceiling effect limits its ability to differentiate among patients with high self-efficacy.^10^ Our study population exceeded a mean score of 10, suggesting that these limitations might affect our study outcome. It may not have been the optimal measurement tool for this study population. Although the difference is higher than the MIC of −0.5 for high-scoring populations, the absence of the MDC makes the interpretation difficult. However, knowing this tool has an evident ceiling effect, a 1.25-point difference on a 0 to 12 scale can be seen as clinically meaningful.

The MDC for the Pain Disability Index (PDI) is higher than the observed difference between our pain groups. However, the MIC is close to our observed difference, with a discrepancy of only 0.07 points. These findings are based on a population with a higher level of disability than ours.^23^ The reference PDI value for patients with painful musculoskeletal and spinal disorders is 37.8 ± 14.2 (5.4 when divided by 7), much higher than the 0.99 (SD 1.43) for our minimal pain group and 2.31 (SD 1.84) for our moderate to severe pain group.^24^ A bottom effect may influence our study population. Thus, in a population with relatively low disability compared with other subgroups where the MDC and MIC are based, a difference of 1.33 points in our study can be considered clinically meaningful.

The difference in patient concerns exceeds the MDC, although no established MIC exists.^6^ We observed a 2-point difference on a 0 to 10 scale, from relatively minimal concerns (2.61, SD 1.90) in the minimal pain group to moderate concerns (4.49, SD 2.52) in the moderate-to-severe pain group. This evident difference indicates a clinically meaningful difference concerning with higher pain intensity. By contrast, the difference between patients with first-episode and recurrent acute pain is only 0.4 on the same scale, which is below the MDC and can be considered not clinically meaningful.

### 4.1. Strengths and limitations

When treatment beliefs and therapeutic relation variables were adjusted for patients' concerns, age, and sex, the differences between the 2 pain groups tended to get smaller and were less significant. Similarly, after adjusting for various psychological factors in the regression analyses—specifically catastrophizing, depression, and kinesiophobia—the differences between the pain groups decreased. This suggests an interrelatedness and that other factors may influence these variables more than by pain intensity alone. The initial findings of significant differences in the unadjusted results from the T tests might oversimplify more complex interrelations between psychological factors and experienced pain, suggesting that at least a part of the differences can be explained by potential confounding factors. Correcting and further analyzing these differences is crucial and represents a strength of this study, as it aids in demonstrating actual differences between the groups. Notably, after adjustments for various beliefs, psychological factors, age, and sex, the differences in patients' concerns, self-efficacy, and disability remained significant, underscoring the robustness of these findings.

### 4.2. Interrelationships

The potential complex interrelations between psychological factors, illness perceptions, and experienced pain intensity become apparent in the data analysis of this study. These factors are known to often be highly correlated^2^ and/or likely have common underlying, or at least partly overlapping, constructs.^7,25^ Catastrophizing, defined as an exaggerated and negative cognitive–emotional schema activated during actual or anticipated painful stimulation, was originally described as a maladaptive cognitive style prevalent among patients with anxiety and depressive disorder. Catastrophizing and kinesiophobia are closely related, whereas catastrophizing often leads to increased kinesiophobia, suggesting that negative perceptions of pain contribute to a heightened fear of movement.^30^ While catastrophizing is a broader tendency to respond negatively to pain, kinesiophobia specifically focuses on the fear of movements that could exacerbate pain.^30^

Operational and conceptual interrelatedness presents interpretive challenges among these variables. Depression and catastrophizing often co-occur in patients with chronic pain, with catastrophizing more directly linked to the anticipation and experience of pain, whereas depression encompasses a broader range of emotional and affective symptoms.^25^

Significant overlap exists between kinesiophobia and catastrophizing; both are associated with negative emotional reactions to pain and are linked to adverse illness perceptions, suggesting that these cognitions together constitute a domain of negative emotional cognitions. Established relationships between various cognitive concepts have shown that self-efficacy is associated with fear-avoidance cognitions and catastrophizing in individuals with chronic pain.^9,29,7^

Despite self-efficacy, cognitive coping styles, fear-avoidance cognitions, and illness beliefs being considered theoretically distinct entities, empirical evidence and theoretical similarities suggest considerable overlap among these concepts. In clinical practice, the interaction between catastrophizing, kinesiophobia, distress, depression, self-efficacy, and illness beliefs must be taken into account when interpreting patients' clinical presentations and exploring treatment possibilities and limitations.^18^ It is expected that not only 1 factor shows higher scores.

Considering this, whether we categorized the different variables in the correct models for regression analyses can be questioned. Psychological factors and illness perceptions are interrelated.^29^ Consequently, the interrelated effect of factors in different models can be overlooked, potentially obscuring the true differences between the 2 pain intensity groups.

### 4.3. Research implications

In our earlier prognostic study, we used NPRS 3, a common cut-off point, to identify patients in the chronic pain group.^31,32^ However, the minimal differences we observed raise questions about the correctness of this threshold.^1,14,36^ Its arbitrary nature suggests that alternative thresholds might yield different outcomes. Moreover, this study highlighted that pain of low intensity is correlated with lower disability and psychological impact, contrasting with chronic pain patients who often exhibit higher scores in these areas.^12,17,35^ This underscores the need for further research to establish a more clinically relevant cut-off point that could inform clinical research and the WHO's guidelines for diagnosing CPP, which currently recommends including NPRS scores for pain, disability, and distress without specifying a mandatory threshold.^19^

The ICD-11 offers a more specific definition of chronic pain; however, it still permits considerable variability in how researchers apply this definition, as most studies primarily focus on pain intensity. This flexibility can lead to subjective patient categorization in clinical studies, potentially introducing biases in outcome measures. Clearer guidance on pain intensity thresholds and the classification of recurrent pain are essential.

Establishing an evidence-based cut-off point for pain intensity that considers its interference with emotional well-being and disability, along with sustained pain above a specific threshold, could improve the accuracy of inclusion criteria and outcome measures. This would exclude recurrent pain cases with minimal pain between flare-ups, improving the precision of chronic pain classifications and the validity of research findings.

## 5. Conclusion

There are no significant or clinically meaningful differences in clinical characteristics or pain recovery rates between a first-episode pain period and pain in a recurrent patron in NSNP. Significant differences exist in the impact of pain severity on daily activities, patient concerns, and self-efficacy. We considered the differences as clinically meaningful.

## Disclosures

The authors have no conflict of interest to declare.

## Appendix A. Supplemental digital content

Supplemental digital content associated with this article can be found online at http://links.lww.com/PR9/A291.

## Supplementary Material

SUPPLEMENTARY MATERIAL
