# Supplementary material for: First-episode vs recurrent nonspecific neck pain: clinical characteristics, recovery, and the impact of pain severity on well-being and functionality
Source: Pain Rep. 2025 Apr 3;10(3):e1259. doi: 10.1097/PR9.0000000000001259 (PMC11970887; doi:10.1097/PR9.0000000000001259)

Appendix 1 Anatomic region neck pain[37]

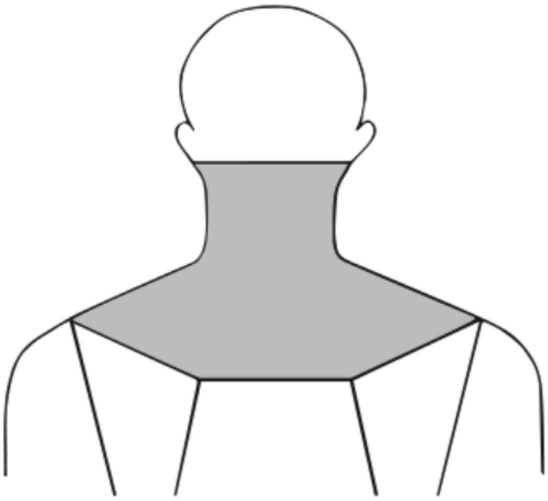

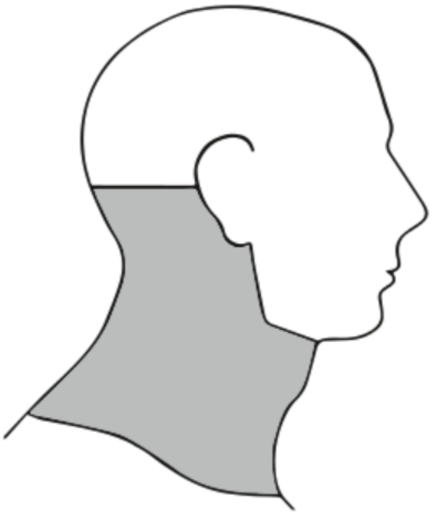

Appendix 2 Variables and their measurement method

Variables	Measure and Range of the Scale	Hypothesis
**Patients' characteristics**		
Sex	Self-report question (Male / Women)	1
Age	Self-report question	1
Work status	Self-report question (Yes/No)	1
Education	Self-report question different education levels.<br><br>Categorized in low level and high level of education.	1
**Symptoms**		
Pain intensity at baseline	Numeric Pain Rating Scale (NPRS) Range 0-10 "On a scale of 0 to 10, how much pain do you experience? Where 0 is no pain at all and 10 is the most imaginable pain"	1
Duration of neck pain	Number of weeks	1
Reported pain in different body regions	Self-report question:<br><br>Do you also experience pain in other parts of your body? (yes/no)	1
Accompanying headache	Self-report question: Have you experienced accompanying headache(s) since you have neck pain?<br><br>Yes / No/ I had headache(s) before the neck pain.	1
Disability	Pain Disability Index (PDI) is a 7-item Pain Disability Index (PDI) is a 7-item questionnaire to investigate the magnitude of self-reported pain-related disability. The PDI measures family/home responsibilities, recreation, social activity, occupation, sexual behavior, self-care, and life support. Higher scores indicate higher interference of pain with daily activity.<br><br>The sum score will be divided by the entered items (range of 0-7)	1 and 2
**Lifestyle factors**		
Physical activity	Measured by the activity level according to the Dutch Healthy Exercise Norm.<br><br>Dived into three categories:<br><br>(1) I don't move 30 minutes any day a week of moderate intensity.<br><br>(2) I'm exactly in between one and three<br><br>(3) I am five days or more active per week	1
Smoking	Self-report question: Do you smoke? (Yes/No)	1
Alcohol	Self-report question: Do you drink alcohol? (Yes/No)	1
BMI	Self-report question: What is your height? And what is your weight?<br><br>Body Mass Index (BMI): weight/(length x length in meters)	1
**Sleep quality**		

Sleep Quality	Adjusted sleep quality question from the Neck Disability Index (NDI) and is subdivided in 4 domains; (1) wake up rested, (2) number of hours disturbed while sleeping, (3) fall asleep, and (4) personal experience sleep quality<br><br>(1) Yes / No<br><br>(2) 0-5 Higher scores indicate more hours disturbed while sleeping<br><br>(3) Yes / No difficulty falling asleep<br><br>(4) Yes / No personal experience difficulty sleeping or falling asleep<br><br>If all questions are answered with a negative result the participant is indicated with no sleeping problems (wake up rested, no hours disturbed sleeping, no problems falling asleep and experience no sleep problems). If one question is answered positive, the participant is indicated with sleeping problems.	1
**Psychological and behavior factors**		
Catastrophizing	Pain Catastrophizing Scale (PCS) short version is a 6-item questionnaire that assesses catastrophic thoughts or feelings associated with the experience of pain. Range 0-24. Higher scores indicate more catastrophic thoughts.	1 and 2
Depression	Depression Anxiety Stress Scale 21-item version (DASS-21) Range 0-21, higher scores indicate a higher degree of depression.	1 and 2
Kinesiophobia	Tampa Scale for Kinesiophobia (TSK) 11-item version. Range 11-44, higher scores indicate a higher degree of kinesiophobia.	1 and 2
Distress	Depression Anxiety Stress Scale 21-item version (DASS-21) Range 0-21, higher scores indicate a higher degree of stress.	1 and 2
Hypervigilance	Pain Vigilance Awareness Questionnaire (PVAQ) Range 0-80, higher scores indicate a higher degree of vigilance.	1 and 2
Self-efficacy	Pain Self-efficacy Questionnaire 2-item version. Range 0-12, higher scores indicate a higher degree of self-efficacy.	1 and 2
Coping	Pain Coping Inventory (PCI) is a 33-items questionnaire and is subdivided into six scales: pain transformation, distraction, reducing demands, retreating, worrying, and resting Transforming the classification into an active (pain transformation, distraction and reducing demands) and passive coping strategy (retreating, worrying, resting).<br><br>Active coping = 12-48<br><br>Passive coping = 21-84	1
**Patients' beliefs**		
Duration beliefs	Brief Illness Perception Questionnaire-Dutch language version (IPQ-DLV).<br><br>How long do you think your neck pain will continue? (0 a very short time – 10 forever) Range 0-10, higher scores indicate a maladaptive illness perception.	1
Concerns	Brief Illness Perception Questionnaire-Dutch language version (IPQ-DLV).<br><br>How concerned are you about your illness? (0 not at all concerned – 10 extremely concerned). Range 0-10, higher scores indicate a maladaptive illness perception.	1 and 2
Treatment beliefs	Brief Illness Perception Questionnaire-DLV.<br><br>Single question: How much do you think your treatment can help your neck pain? (0 not at all - 10 extremely helpful) Range 0-10, a lower score indicates a maladaptive illness perception.	1
Therapeutic relation	Self-report question: How much trust do you have in your healthcare provider/ physiotherapist? 0 no trust at all – 10 very much confidence	1 and 2

	Range 0-10	
Identity beliefs	Brief Illness Perception Questionnaire-DLV<br><br>Single question: How well do you feel you understand your illness? (0 don't understand at all - 10 understand very clearly). Range 0-10, a lower score indicates a maladaptive illness perception.	1 and 2

Appendix 3 Flow-chart

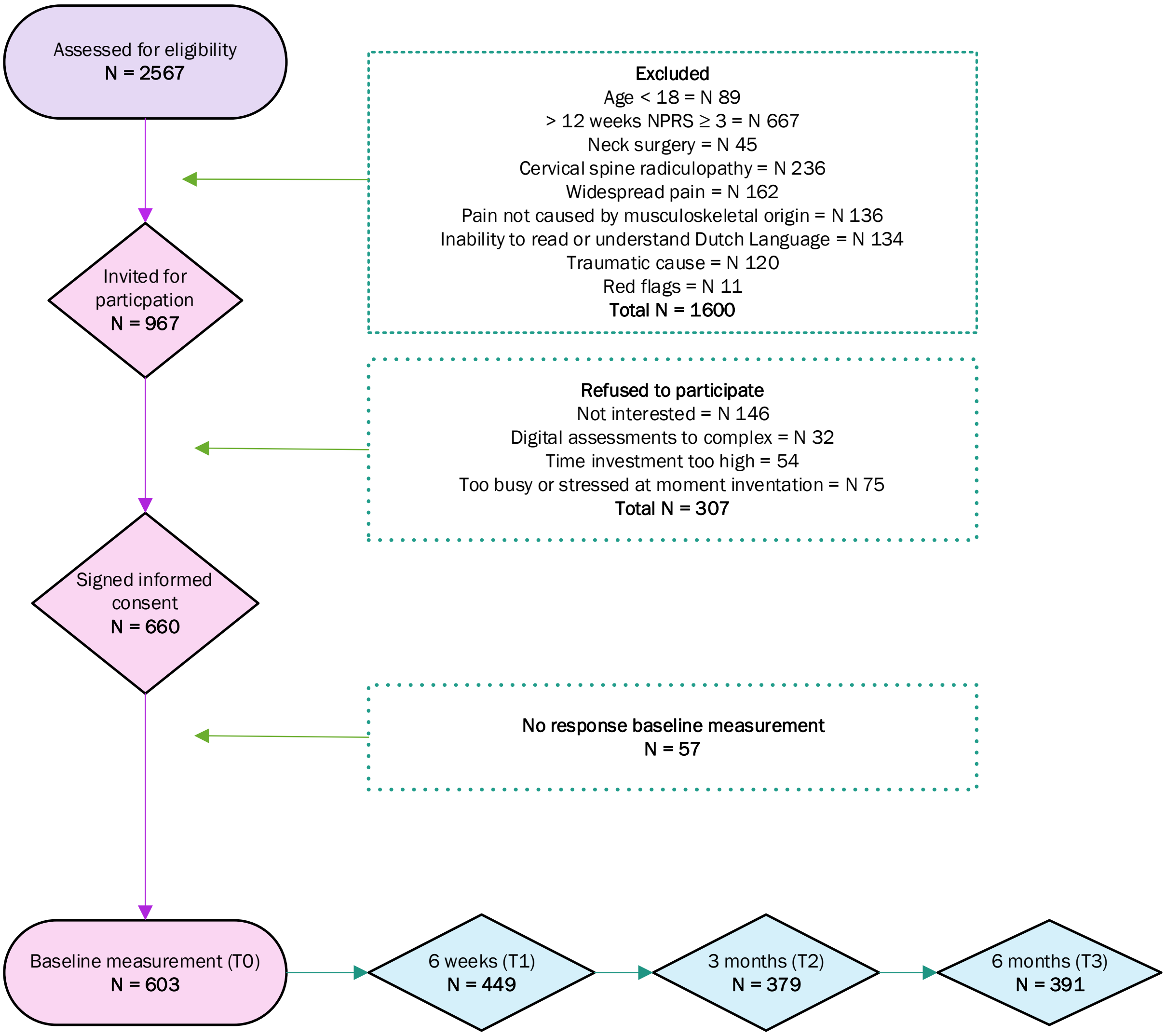

Supplement: SUPPLEMENTARY MATERIAL [file painreports-10-e1259-s001.pdf]
